# Editorial: Menstruation: Myths, mechanisms, models and malfunctions

**DOI:** 10.3389/frph.2023.1158317

**Published:** 2023-04-03

**Authors:** Fiona L. Cousins, Philippa T. K. Saunders

**Affiliations:** ^1^ The Ritchie Centre, Hudson Institute of Medical Research, Clayton, VIC, Australia; ^2^ Centre for Inflammation Research, Institute of Regeneration and Repair, the University of Edinburgh, Edinburgh, United Kingdom

**Keywords:** endometrium, menstruation, endometriosis, fibroids, scarless repair, abnormal uterine bleeding, heavy menstrual bleeding

**Editorial on the Research Topic** Menstruation: Myths, mechanisms, models and malfunctions

## Introduction

The endometrium is a remarkable, resilient, hormone-dependent tissue that prepares each month for the arrival of a blastocyst and a pregnancy. If no pregnancy occurs, endometrial tissue surrounding the uterine cavity breaks down during menstruation releasing tissue fragments, blood, and fluid into the lumen. The appearance of “blood” in the vagina is the hallmark of menstruation, and in a modern society with low birth rate, may occur 400 times during a woman's fertile, reproductive life. During menstruation the endometrium resembles a bloody wound (1) with a strong inflammatory response (2).

The appearance of blood in vaginal fluids has been linked to many societal and religious taboos: there are still large gaps in our knowledge about the mechanisms that regulate menstruation and how their dysregulation contributes to pathologies that have a huge impact on the quality of life of women.

The aim of this research topic was to bring together a diverse range of contributions spanning a wide range of topics from models to societal impacts. The topic contains thirteen papers which fall into three broad categories: Societal Attitudes, Mechanisms and Models, and Disorders (abnormal bleeding and endometriosis).

## Societal attitudes to menstruation

In their fascinating original study entitled “*Menstrual Hygiene Management—Knowledge, Attitudes, and Practices Among Female College Students in Bhutan*” Tshomo et al. have focused on the challenges of menstrual hygiene management faced by young people in countries classified as having a ‘lower middle income economy’ by the World Bank (https://www.worldbank.org/en/country/bhutan/overview). They analysed the data from a self-administered questionnaire completed by just over 1,000 participants. Half of the participants reported their daily activities were affected by menstruation and a quarter missed time at college due to painful periods. It was striking that challenges included lack of access to proper handwashing facilities (soap/water; 80%). The authors hope their findings will inform government initiatives to improve the lives of women and girls. This study was nicely complemented by one conducted in college students in the United States, a rich first world country. Gruer et al. set out to see what strategies were useful in addressing issues of menstrual equity and period poverty in their study entitled “*Menstrual Equity Initiatives at USA Universities: A Multiple Case Study of Common Obstacles and Enabling Factors*”. They focused on Universities undertaking free menstrual product initiatives to conduct a qualitative study finding that, although all had been successful, they varied in terms of implementation strategy with limitations imposed by resources. There are clearly opportunities to share ideas from different countries and initiatives with regard to scale and funding—it is to be hoped that more countries take the same step as the Scottish government who passed a law in 2021 requiring local authorities and education providers to make period products obtainable free of charge for anyone who needs to use them (https://www.gov.scot/publications/period-products-free-provision-scotland-act-2021-equality-impact-assessment/).

## Mechanisms and models

The endometrium is a dynamic and multicellular tissue and in their review, entitled “*Genetic Regulation of Transcription in the Endometrium in Health and Disease*”, Mortlock et al. reviewed the similarities and differences in endometrial gene expression with other body tissues and the role of specific genes in the risk of developing endometrial diseases. Their paper is an excellent primer for anyone interested in improving their understanding of complex genetic methods and concludes with a plea that researchers and clinicians must consider an individual's genetic background when investigating and managing fertility and disease.

Menstruating mammals share a number of different features including spontaneous ovulation and fertility that is associated with an endometrium which has been transformed into a “receptive” state (3). The review by Muter et al. focusses on the importance of the spontaneous differentiation (decidualization) of the endometrial stromal cells that occurs in response to high concentrations of progesterone produced by the ovary following ovulation. This advance in our understanding of the existence of different subpopulations of decidual cells has highlighted mechanisms that may explain risk of miscarriage and offer new therapeutic targets to improve pregnancy outcomes.

The endometrium is almost unique amongst adult tissues in its ability to heal without forming a scar or fibrotic tissue in response to the endometrial “wound”: several of the papers in this special issue focused on the latest evidence related to the mechanisms that are implicated in endometrial repair during the menstrual cycle (Salamonsen Cousins et al. Bellofiore et al.).

Salamonsen reminds the reader that during the shedding of the inner (luminal) surface of the endometrium the tissue is bathed in menstrual fluid which contains live cells, as well as activated leukocytes, soluble cellular components and extracellular vesicles. She highlights the evidence from cell culture and skin and pig wound models that “*Menstrual Fluid Factors Mediate Endometrial Repair*”. Notably she argues that the analysis of this fluid may provide much needed new insights that could be applied to the treatment of poorly repairing skin wounds which are an increasing problem in old age (4).

In two complementary reviews Kirkwood et al. and Bellofiore et al., and their colleagues in Scotland and Australia respectively, review the data on “menstruation” generated using laboratory and Spiny mice and highlight how they have informed our understanding of the basic mechanisms responsible for endometrial shedding and repair. Kirkwood et al. remind the reader that the mouse endometrium does not normally experience shedding and review the refinement of methods that have been applied to laboratory mice to recapitulate the main features of human menstruation including rapid breakdown, hypoxia, shedding and repair as well as the advantage of using genetically manipulated mice for these studies. They have recently followed up on these studies with new data showing transformation of mesenchyme cells into epithelium may complement other mechanisms including epithelial cell proliferation (5). The discovery of naturally occurring menstruation in the Egyptian spiny mouse (*Acomys cahirinus*), a species that also exhibits scarless healing of skin (6), has led to intense interest in the potential of this model species “to Build a Bridge from Bench to Bedside” and improve translation of laboratory studies into clinical therapies for endometrial disorders including heavy menstrual bleeding. The authors discuss insights from studying cycle variation between individual animals and how this might assist in better understanding of vascular remodelling successful implantation.

Following endometrial repair, which occurs in a hormone-depleted environment, the inner layer of the endometrium (the ‘functionalis”) grows rapidly from the basal (unshed) portion of endometrium in response to rising concentrations of oestrogens in the blood. This regenerative capacity of the endometrium is attributed to the “Endometrial Stem/Progenitor Cells” which occur in both the epithelial and stromal compartments. Cousins et. al. provide a comprehensive review of the markers used to identify putative progenitors, their identity, location and hierarchy across the menstrual cycle (Cousins et al.).

## Endometrial disorders

The last group of papers in this special issue consider different aspects of endometrial function that might contribute to its malfunction and how these changes can be used to better understand and treat disorders that have an impact on the quality of life of millions of individuals. Three of these papers are focused on abnormal uterine bleeding (AUB) which can include abnormal frequency as well as prolonged and heavy bleeding (HMB) (Chodankar et al. Watters et al. Uimari et al.) whilst two are on endometriosis (Kuan et al. Mbuguiro et al.).

One of the barriers to improving the management of menstrual symptoms has been inconsistency in terminology which has created considerable confusion. In their article “*Historical Perspectives and Evolution of Menstrual Terminology*” Chodankar et al. give a comprehensive overview of the history and evolution of terminology. The paper has a useful figure showing the timeline of the relevant publications and meetings which have resulted in two internationally accepted classifications under the banner of the Federation of Gynecology and Obstetrics (FIGO). The paper by Watters et al. considers current understanding of endometrial physiology at menstruation highlighting the contribution of the specialised endometrial vasculature and coagulation system. They use these insights as a platform for better understanding of gaps in knowledge and what is known about aberrations in endometrial physiology that can cause symptoms of AUB, concluding with an ideal model for management of AUB that includes consideration of patient preferences. One of the causes of AUB identified in the FIGO classification system is the presence of uterine fibroids (Leiomyomata): in their review, Uimari et al. remind the reader that in more than half of patients these benign growths cause HMB, pelvic pain or infertility (Uimari et al.). They consider the treatment options available for fibroids (and symptom relief) and the current theories about the link between disordered vasculature architecture and/or vasoactive growth factors and the increased incidence of HMB in this patient group.

Endometriosis is estimated to occur in ∼10% of women of reproductive age: symptoms can begin early in adolescence and can be debilitating (7). In their review Kuan et al. consider how “*Menstrual Dysregulation*” can contribute to the pathogenesis of endometriosis which is associated with the occurrence of tissue “lesions” resembling endometrium in sites outside the uterus, most often in the peritoneal cavity. Their review highlights some parallels with other endometrial disorders such as AUB including dysregulation of inflammatory factors and the potential use of menstrual fluid as a source of biomarkers complementing the information in the review by Salamonsen. One of the challenges faced by patients with endometriosis is the time taken for those experiencing symptoms to get a diagnosis which is ∼7 years on average. This is in part reflects the lack of robust and reproducible diagnostics that do not depend on imaging or surgery. Mbuguiro et al. make the case for the application of “*Computational Models for Diagnosing and Treating Endometriosis*” by considering three computational modelling approaches that have been used and how each approach (regression, pharmaco- kinetics/dynamics and quantitative systems pharmacology) can answer different questions about endometriosis. This paper is particularly useful for the non-expert as they summarise the mathematics involved, the benefits and limitations of each model and how we might combine these approaches in the future.

## Conclusions and prospects for future studies

These papers highlight how different approaches and resources have shaped and informed our understanding of menstruation and endometrial disorders. They offer a unique resource to people wishing to learn more about access to resources, endometrial function and malfunction. In addition, the potential of this information to inform improved diagnostics and therapies for disorders such as AUB and endometriosis is considerable and a better understanding of menstruation may offer unique insights into mechanisms of repair without fibrosis.
